# Key Challenges and Future Directions When Running Auditory Brainstem Response (ABR) Research Protocols with Newborns: A Music and Language EEG Feasibility Study

**DOI:** 10.3390/brainsci11121562

**Published:** 2021-11-26

**Authors:** Efthymios Papatzikis, Mahmoud Elhalik, Shannaiah Aubrey Mae Inocencio, Maria Agapaki, Rosari Naveena Selvan, Faseela Shejeed Muhammed, Nazreen Abdulla Haroon, Swarup Kumar Dash, Maria Sofologi, Antonia Bezoni

**Affiliations:** 1 Department of Early Childhood Education and Care, Oslo Metropolitan University, 0167 Oslo, Norway; 2 Neonatal Intensive Care Unit, Latifa Women and Children’s Hospital, Dubai 9115, United Arab Emirates; msElhalik@dha.gov.ae (M.E.); FHVMuhammed@dha.gov.ae (F.S.M.); nazreenpharoon@gmail.com (N.A.H.); sDash@dha.gov.ae (S.K.D.); 3 Department of Psychology, Canadian University Dubai, Dubai 415053, United Arab Emirates; ishannaiah@gmail.com; 4 Independent Researcher, 71305 Iraklio, Greece; maria.agapaki94@gmail.com; 5 Institute for Physics 3—Biophysics and Bernstein Center for Computational Neuroscience, University of Göttingen, 37073 Göttingen, Germany; rselvan@uni-muenster.de; 6 Department of Psychology, University of Münster, 48149 Münster, Germany; 7 Psychology Laboratory, Department of Early Childhood Education, School of Education, University of Ioannina, 45110 Ioannina, Greece; m.sofologi@uoi.gr; 8 Institute of Humanities and Social Sciences, University Research Centre of Ioannina, 45110 Ioannina, Greece; 9 Department of Midwifery, Røyken Health Station, 3440 Røyken, Norway; toniampez@gmail.com

**Keywords:** infants, newborns, auditory brainstem response, ABR, EEG, brainstem, development, music, language, feasibility

## Abstract

Although many musical intervention studies exist in the wider framework of neuroscience and psychology, the preliminary importance of feasibility studies is rarely discussed. Adding to this fact the limited research existing on the therapeutic and restorative potential of music exposure during early developmental periods, pushed us to concentrate on investigating newborns’ perception of music and its impact on the brain. Here, we explore the feasibility of a randomized controlled trial (RCT) approach when measuring and comparing the neurophysiological perception of music versus language on the brainstem of newborns using auditory brainstem response (ABR). Twenty-five healthy full-term infants were recruited, eight of which were measured within their first 10 days postpartum. The evaluation of the study’s feasibility appealed to five main objectives that essentially answer the question: Can our protocol work? Each objective proposes questions based on Orsmond and Cohn’s guiding framework, designed to assess, and assist feasibility in understanding barriers toward a study’s success. Our results justify that newborns are well capable of undergoing the study and given meticulous considerations and improvements on the intervention resources. The procedure’s communication and technical obstacles are resoluble. Moreover, assimilation of external factors to adapt, such as the culture variation and the ABR protocol implementation are necessary. The study was well received in the selected region (Middle East), and the recording procedure showed potential outcomes for a comprehensive RCT.

## 1. Introduction

Although a large number of musical intervention studies exist in the wider framework of neuroscience and psychology (for example see reviews [1,2,3,4,5,6,7]), the preliminary importance of feasibility studies is rarely discussed—and consequently used—by researchers in the specific field. In general literature, the terms ‘pilot’ and ‘feasibility’ have been used interchangeably [8] as according to [9] both of these study designs assess the potential for successful implementation of the proposed main intervention. Indeed, both study types are critical [10] to produce reliable and valid findings while facilitating the formation of enhanced study designs. However, it has been suggested that feasibility studies are better suited to make us understand the challenges in the implementation of newly constructed intervention tools, while enabling us to more reliably bring a research design into the field [11]. According to the National Institute of Health Research [12] pilot studies are ‘smaller versions of the main study used to test whether the components of the main study can all work together’, whereas feasibility studies focus on conducting novel research and specifically examine whether a study can be done and fitting to the area of application. The distinctive feature of feasibility studies, therefore, focuses on the process to answer the question “can it work?”, whereas the distinctive feature of pilot studies focuses on the outcome “does the intervention show promise?” [13]

Considering the above, we embarked on testing the feasibility of our novel study concept referring to a randomized control trial (RCT) approach on newborns brain development through music. According to our study design, our prospective aim is to measure the neurophysiological responses to perception of music versus language on the brainstem (auditory brainstem response—ABR) of at least 120 healthy full-term newborns in the first 10 days postpartum. Previous studies on the brainstem and sound or music interdisciplinary domain have discussed in detail the processes, advantage, and success of the auditory brainstem response technique in several other applications in the field [14,15,16]. More so the benefits of the ABR technique in newborn populations [17,18,19]. Considering the fact that for music has been generally shown to be a positive influencing factor of growth and resilience in the human population [20,21,22], we decided to specifically focus on the developmental domain, and we hypothesize that a more efficient functional brainstem development will be projected in the first two postnatal weeks of life of healthy newborns, when music is used as an intervention tool, following a predefined set of music stimuli, and compared to storytelling and no intervention at all. Our hypothesis is drawn upon prior research showcasing on the one hand music’s research advantage at these first stages of development—even in the most sever of environmental or clinical situations—[23,24,25,26] and on the other hand, that music may be an equally or perhaps more informative stimulus than language for brain development [27,28,29].

The feasibility stage we report here, employed a well-defined set of objectives (please see Section 2.3 below) to pinpoint and extract valuable data to consider while applying our protocol, and aimed to understand the extent to which our research design is able to produce viable results, while perceiving, documenting and analyzing in detail the changes that can or should be made in the data collection process, and consequently suggest a better implementation of our intervention and measurement tools. By completing and reporting this stage of our work, we not only hope to solidly improve our own research practice in this quite uninformed and under-researched, yet very sensitive and important field of the newborns’ brain development through music and sound, but to also provide an informative basis for other researchers to reflect and rely on for the benefit of their own research endeavors in this domain of inquiry.

## 2. Materials and Methods

### 2.1. Sample

Our feasibility study was conducted in the Newborn/Labor ward in Latifa Hospital Dubai, a tertiary care referral hospital in the United Arab Emirates (UAE). The study was approved by the Dubai Health Authority (DHA) (authorization number: DSREC—07/2019_22). The feasibility sample consisted of 25 randomly selected newborns and their mothers (dyads). All of them had Arabic as their native language. Unfortunately, due to the COVID-19 pandemic outbreak, our recruitment and data collection process had to abruptly stop, limiting ourselves to a smaller sample size compared to our initial expectations.

Each recruited dyad consisted of a healthy mother and her newborn (≥37 weeks gestation). All recruited newborns had an APGAR score +8 while they had successfully passed an Otoacoustic Emissions (OAEs) test before being accepted in the study. Infants passing through labor or postpartum complications (for example hypoxic events or high levels of bilirubin in need of photo-treatment) were excluded. We received informed verbal acceptance and written consent by all mothers participating in this feasibility study, while we also took care to not interfere or obstruct any routines and postpartum protocols prescribed by the gynecologist and the midwife. All written consent material was distributed in English as well as in Arabic, while verbal and written occasional guidance communication took place either in English or Arabic, as needed per case.

### 2.2. The Study Design

The newborns were randomly divided into two experimental groups, following a music and a storytelling intervention. No control group could be recruited at this stage as originally designed, due to the COVID-19 outbreak as mentioned above. Both experimental groups were ABR measured pre and post the intervention. Starting right after the first 24 h after delivery, the intervention was taking place for seven consecutive days, twice per day during any two of the daily breastfeeding/feeding episodes. For the music experiment group (MEG), we used two purely instrumental musical pieces coming from the classical era by Mozart and Haydn. The use of classical music was based on the assumption that this type of music was not typically accessible to newborns in the region to be exposed to, hence reducing the degree of exogenous to the protocol exposure as a confounding variable. The two music pieces were professionally recorded and performed by musicians. Their overall duration was 20 min. The pieces were compressed to an mp3 high bit-rate format for easier distribution through a mobile phone application. They were pre-processed and re-mastered to normalize and equalize their sound output and their frequency distribution below and up a certain frequency threshold (High Pass: 15 Hz—Low Pass: 8 kHz) to achieve sound consistency for all mobile phone speakers. They were also balanced at a 120-bpm rate.

For the storytelling experiment group (SEG), the Pinocchio fairy-tale (English version; duration 20:17 min) was used as the counterbalancing intervention tool. As in the music stimuli above, we used an English based fairy-tail stimulus to avoid prior language training and habituation confounding effects. The fairy-tale was recorded anew in an anechoic chamber by a male voice-over specialist while was digitally pre-processed and mastered to mirror the sound characteristics and distributing mode of the music intervention tool. Our aim through this specifically designed re-mastering and distribution process was to achieve a controlled profile of intervention, excluding to the best possible level confounding variables which could impact our final results. The pre-processing for both the music and the fairy-tale took place on the Sonic Visualizer software platform [30]. In regard to the distribution and application of both intervention tools during the breastfeeding/feeding episodes at the hospital or at home, all mothers were instructed to use their mobile phone as the sole sound production unit, always using 60% of the total volume capacity of the device. This precaution was taken to avoid excessive acoustical stimulation. The mothers were also instructed to place the mobile no more or less than two to three meters away from the infant.

As far as the pre and post-intervention ABR recordings are concerned, they ranged from 30 to 180 min. During the measurement process, the infant participant was placed on a comfortable baby bassinet or the lap of their mother (preferably when asleep) and was fitted with 4 electrodes. These EEG electrodes were placed on the infant’s scalp on the high forehead at the Cz location (ChnA and ChnB (+)/non-inverting), on the Fp1 and Fp2 regions, on the M1 and M2 regions (i.e., right and left mastoids; ChnA and ChnB (−)/Inverting) below the A1 and A2 regions (earlobes), as well as on the lower forehead (G region; ground). During the ABR recording, we used insert earphones, and applied binaural—non-simultaneous—stimulation for both the pre- and post-intervention phases in order to maximize data collection, to cross-reference electrical signals and recording results, and ultimately avoid as much as possible missing data. The brainstem response was induced by a ‘clicks’ derived stimulation protocol (0.1 ms duration; 27.7 p/s rate; 1024 sweeps) applied at 65 dBHL as described in the British Society of Audiology protocols for early years screening and neurodiagnosis [31]. With our ABR recorded data analysis we aimed to statistically control the Δ of the peak V latencies pre and post intervention as well as the Δ of the main inter-peak (V–III, V–I) latencies pre and post intervention for the averaged signal of each group. Defined by positive developmental ABR trends as described in the relevant literature [32], our analysis would lead us to confirm a well applied ABR measurement and analysis protocol.

### 2.3. The Feasibility Analysis

By running this preliminary study stage, our goal was to check its overall feasibility profile. Therefore, we tried to answer the general question “Can it work in this specific clinical environment, using these specific tools and design, considering the existing clinicians’ training as well as the participants’ beliefs and prior experience—or lack of it—with similar research projects?”. To achieve our goal, we answered follow-up questions based on a previously published health-interventions feasibility testing theoretical framework constructed by Orsmond and Cohn [13] (for a complete list of the questions we used, please see Appendix A). These follow-up questions were categorized in five overarching objectives: evaluation of recruitment capability and resulting sample characteristics (Objective 1), evaluation and refinement of data collection procedures and outcome measures (Objective 2), evaluation of acceptability and suitability of intervention and study procedures (Objective 3), evaluation of resources and ability to manage and implement the study and intervention (Objective 4), and preliminary evaluation of participant responses to intervention (Objective 5).

For Objective 1, it was important to determine if we recruited appropriate participants representative of the target study population, and if our proposed intervention was related to them. To address this objective, we examined the recruitment rates (the number of participants that entered the study, the duration of the recruitment, the refusal rates for participation, and possible obstacles to recruitment), the eligibility criteria (clearness and sufficiency of the criteria, the number of eligible members of the targeted population that are accessible in the local community), and the relevance of the intervention to the intended study population, i.e., if study participants showed evidence of need for the intervention and if participants’ characteristics were consistent with the range of expected characteristics based on research literature.

Regarding Objective 2, it was important to determine the appropriateness and suitability of the data collection procedures and outcome measures for the intended population and purpose of the study, so as to confirm that we will be able to interpret our findings during the full range randomized controlled trial (RCT) study. To address this objective, we answered follow-up questions about participants’ ability to complete the measures, appropriateness of the amount of data collection (competence to complete the data collection procedures, entailment or not of a reasonable amount of time to the overall data collection plan), and appropriateness of the measures for the specific population and intervention based upon prior studies in the research literature.

For Objective 3, it was important to determine if the study procedures were acceptable to the participants. To address this objective, we examined the retention and follow-up rates, the adherence rates, intervention attendance and engagement (intervention’s appropriateness with the daily life activities of study participants, proper time, and participants’ ability to complete the intervention, acceptability and satisfaction of the intervention to participants), and safety and unexpected adverse events of the procedures in the intervention.

With Objective 4, we aimed to determine if the research team had sufficient resources and ability to successfully manage the study, to prove that the investigators were able to manage the proposed project and subsequently conduct a larger RCT study. To address this objective, we answered follow-up questions about whether the research team had the administrative capacity, expertise, skills, space, and time to conduct the study and intervention, ethics in implementing the study (compliance of research staff with the approved participants’ protocol, reporting of adverse events during the study), budgetary considerations, and technology, equipment needs and training concerning collection, management, and analysis of data.

Finally, Objective 5 was about determining whether the study showed promise of being successful with the intended population. To address this objective, we examined the quantitative and qualitative data at our disposal (showing possible and expected changes in key outcome variables; whether the intervention had promise based on the estimates of effects, etc.). In the case of a not promising methodology, we got ready to re-examine the appropriateness of the data collection procedures and outcome measures for the particular population and study, possible evidence of the implementation of intervention in a non-intended and non-changeable manner towards the desired outcomes, the number of adaptations in the intervention process to assess the participants’ results, and congruence of findings with the available prior relevant literature.

## 3. Results

### 3.1. Evaluation of Recruitment (Objective 1)

We collected our data in one of the most prominent hospitals in Dubai. This clinical site deals everyday with many cases of high-risk pregnancy [33]. According to the hospital’s annual report of pregnancies in 2020, approximately 1900 of them were identified as high-risk. This meant that almost 50% of the available sample had to be excluded from participation. Based on the approximate number of pregnant mothers received annually, monthly, weekly, and daily (Table 1), an average of 2 participants were finally recruited per day out of the 10–11 available ones (reasons will be analyzed below). This recruiting pace provided us with an estimate of 12–15 weeks to reach a plausible sample of a hundred participants at a refusal rate of 50–60% out of the potential day sample who were available and fitted to our criteria.

Although we realized that it was slightly challenging to recruit many participants into our study, we finally agreed that the eligibility criteria were very clear and sufficient to achieve our goal—not too inclusive nor over-restrictive without a reason. In the timeframe we ran our feasibility study, we managed to recruit 25 participants. The participants were randomly assigned to either the music (MEG; 12 infants) or the story (SEG; 13 infants) cohort. However, only eight of them (MEG: 4 infants; SEG: 4 infants) managed to complete both pre- and post-intervention measures—that is, 32% follow-up rate.

#### 3.1.1. Obstacles to Recruitment

We identified three main obstacles for the low enrollment rates. More specifically, the first one concerned the collaborating research staff (CRS—lead doctors and clinical staff) involved in the recruitment procedure. Despite the enthusiasm and appropriate assistance of the CRS in the delivery ward, we realized that after running the protocol for a couple of days, they were still not familiar enough with the procedures involved in the study. As appeared through in-team discussions, doctors’ unfamiliarity with study procedures was because they were not proficient with the project’s content and logic, despite running a fully loaded training session with them before applying the protocol in the field. Many considerations were put in place from their part in relation to music, religion, and culture, while also being unable to answer related questions coming from the participants’ side. To remedy this problem, the principal investigator (PI) asked permission—which was granted—from the hospital’s administration to be present at all times during the initial phases to aid the recruitment procedure. The PI’s presence during recruitment—answering real-time questions on content and procedures—provided the CRS a further opportunity to learn more about the existing knowledge on the brainstem’s interaction with music and sound, and on its potential impact on newborns, bringing thereafter a better sense of content knowledge and consequently achievement towards recruitment.

The second obstacle was related to participants’ ineligibility and refusal. Our explanation for this setback entailed five different arguments. To be more precise, three of these arguments were possible reasons why the participants did not come back. The first one was negligence or change of interest in the study. We remedied this one by revisiting the involvement of the participants in the study two or three times before recruiting them to be sure about their perception on the study’s processes as well as their motivation. The second reason was the difficulty in returning to the hospital setting due to distance. We suggested a remedy to this problem by offering home visits. The third reason was difficulties for mothers in following up newborn routines, hence, transferred difficulty to attend follow-up appointments. We attempted to remedy this one by engaging the research midwife even more than expected. As a result, she started providing personal sessions to participating mothers before leaving the hospital setting, discussing possible issues and solutions to confront problematic follow-up occurrences.

The other two arguments that were evidently limiting our recruitment concerned communication and cultural barriers. Limited communication was creating issues on the expected involvement. For example, some of the parents who were not fluent in English required instruction in their own language, which was not known nor spoken by the main researcher. Therefore, assimilating the recording process outside of the hospital became difficult unless instructions were spoken in Arabic. Another example that proves limitations in communication appeared during a follow-up session. Specifically, during a post-intervention appointment, one parent (Mother 19) expressed misunderstanding of how to deliver the intervention. Hence, we suspected that the procedure was not delivered at all to the participant during the trial stages and so the participant, even though was assigned to the story group during the initial stages of the study, was assigned to the control group. In addition, during the revision of the ABR recordings and pre-processing, the pre-measurement recordings of the participant displayed ambivalent signals, i.e., participant’s recordings were not consistent with each other and were showing different trends despite being collected consecutively. However, the presence of clear signals in the post-measurement prevented us from fully discarding the data, keeping them on hold to decide for their later inclusion in the overall control-group sample. The limitations in communication made it apparent that the researcher must be eloquent and trained in providing the instructions. To minimize the consequences of this problem, we remedied the language barrier by including an Arabic speaking recruitment researcher communicating all material both in English and Arabic at the same time, at all times. Finally, the fifth possible argument for refusal was directly received as feedback and was referring to discordance between parents. In some cases, we had participants where one parent was eager to be involved in the study but the other parent declined. We respected that both parties had the right to decide for their children and must unanimously be willing to be involved. Henceforth, the participants were advised to discuss their participation with their spouses and were given ample time to confirm and sign their letter of consent.

The last obstacle that had put pressure on the recruitment process was the music preferences of the parents which were influenced by cultural inclinations. Quite a few parents had reservations about the music used in the study, stating that it was not appropriate based on their socio-religious background. We remedied this obstacle by either informing them in more detail about the music’s use in the study or by offering them a place in the SEG. We were also reassuring them that their participation would not cause any dilemma nor harm to the child as our methodology prioritizes the well-being of the newborn, stressing the ethical approval granted by the relevant authorities (i.e., DHA).

#### 3.1.2. Relevance of the Intervention to the Intended Population

It has been found that in the first weeks and months of human development, specific stimuli, such as music, delivered in-direct to the baby may provide an efficient platform of advancement when correctly used as intervention tools [34,35,36]. In this context, music can be easily employed as an intervention tool, as it is not clinically invasive. Additionally, we know that a primary brain system related to music’s perception and analysis—the brainstem—is the first to be qualitatively functional in the perinatal period of life. According to some scientists, human auditory activation at the level of the brainstem is evident at 28 weeks of gestation [37]. Others have suggested for an even earlier auditory evoked response to be evident at 16 weeks gestational age [38].

Considering this evidence, taking into account that there is no known literature debating this set of participants’ inclusion criteria and intervention together, and after consulting neonatologists for the project’s potential viability in this very sensitive context immediately after delivery we reached the point to justify the criterion of ‘0–10 days old’ as the most suitable choice of participants for our study. To further establish population relevance, we also evaluated the extent to which new mothers showed evidence of welcoming or even need towards either the intervention or the ABR measurement itself. Despite the lack of known literature—or even anecdote precedents—suggesting otherwise, we were surprised to receive mothers’ will and gratitude to participate understanding that the project may help them comprehend better the functions and features of music related to infants, as well as how to deal later on with it in terms of development.

### 3.2. Evaluation of Data Collection and Outcome Measures (Objective 2)

For the first four recruits, we found that our participants had a slight difficulty to fully grasp the protocol’s concept after only reading the information provided in the consent form—an approach that was initially decided based on the context’s sensitive clinical characteristics. This situation resulted in extended pre- and post-intervention ABR measuring times, misuse of consumables, as well as a lot of lost time trying to secure a solid intervention implementation after mothers’ departure from the hospital. Considering the importance of the situation, and in order to acutely achieve a more effective data collection process, we asked for relevant permissions to physically access the new mothers, right after delivery. We consequently changed our recruitment information delivery mode.

After the fifth recruit, we started explaining to potential participants both orally and in writing the aim, targets, and procedures of the project in the first possible instance after delivery, while we started scheduling in the same day for a follow-up visit to secure willingness to participate. This more ‘aggressive’ approach of recruitment and delivery information seemed to better fill potential implementation gaps coming from the side of the mothers, while leading to improve the protocol’s implementation timings at its later stages, too. Additionally, the whole research team’s efficiency was better fine-tuned after this change, reflecting consequently on better scheduling the recording appointments and saving of consumables. Moreover, the more direct and precise recruitment process helped indirectly to obtain a cleaner ABR signal during the recording sessions as there was freer time for the CRS to pinpoint and relocate certain materials or machinery, or even change certain clinical routines happening in and around the laboratory interfering with our EEG signal while recording (for example relocate the recharging breast milk pumps or alter the room disinfection scheduling of the neighboring rooms).

Finally, we also realized that we had to increase the ABR recording session’s duration. The data collection protocols were designed to ensure that the manner and timeframe of the collection was reasonable to gather meaningful recordings, while most importantly being within the newborn’s best interest. During our feasibility study, there were no reports of distress, while most of the recordings were achieved while the infants were asleep. In this ideal condition, an ABR recording takes 30 min to complete. However, especially for the post-intervention recording appointment, it was not always going as smoothly as expected. In many cases we realized that mothers were deviating from the suggested in-coming for the ABR measurement protocol. Specifically, the parents were advised in advance to feed their infant prior the recording to soothe the newborn to sleep as it was an ideal strategy to minimize the duration of the recording process. Unfortunately, the participants were not always compliant with this advice, causing delays in their recording appointment as well as mild inconvenience to the following to them punctual to our advice and timeframe participants. In order to remedy this potentially negative element, we assigned each recording appointment an additional three hours. According to this change, our overall recording time for each participant was fluctuating from 45 min (mostly happening post-delivery, during the pre-intervention ABR recording session) and up to three and a half hours. This plan helped us to allow ample time for the neonates to be fed if needed, be soothed, and to fall asleep. Although this protocol change allowed only for two appointments of recordings to take place per day, it ensured that the measurements were conducted according to the best possible standards while potentially ‘stressful’ appointment overlap among participants was avoided.

### 3.3. Evaluation of Acceptability and Suitability of Intervention and Study Procedures (Objective 3)

A combined listening-(breast)feeding intervention approach was employed to control for external and excessive nursing factors that could possibly affect the intervention’s accuracy and could not be accounted for through other means. The reasons that led us to follow this path were twofold. Firstly, (breast)feeding seems to be a specific psychobiological ritual for all newborns including mother’s contact with their babies. In this sense, a common state was expected to be formed to all participants during the intervention, while excessive stress or activity was avoided. Secondly, it provides a commonly controllable environment as it is a process that surely takes place every day. Infants are usually (breast)fed at least eight times per day. Our intervention was asking for only two of these times (once in the morning and once in the evening) to use music/storytelling as a stimulus. It was also asked from the (breast)feeding caregivers to initiate the intervention process every time at the same time during the day as much as possible, in a quiet and comfortable place. This way exogenous and distracting factors to the listening process would be minimal. Ultimately, through this design, we realized in practice that we were offering new mothers plenty of opportunities to correctly invest in the protocol, without putting extra effort nor time into it.

Additionally, the intervention proved to be suitable for our intended sample for two specific reasons. The first one was the participants’ mother tongue. Their native language was Arabic. So, we would expect to see a clearer response on the intervention due to the English stimulus. Confounding variables referring to language inhibition or over-usage combined with music training were taken care of this way [39]. The second reason was the cultural background of the participants. People in Dubai usually listen to Arabic pop music, rock music, or traditional music (i.e., Khalil and Bedouin folk music). Therefore, the assumption that the participants are less likely to be exposed to classical music was available in this context. Based on these facts, we believe that we created an intervention platform whereupon we could get clearer, not distorted results by relevant confounding variables, and with a greater power of effect.

### 3.4. Evaluation of Resources and Ability to Manage and Implement the Study and Intervention (Objective 4)

For the most part of our feasibility study, the research team had the administrative capacity, expertise, skills, space, and time to conduct the study and intervention. However, considering the sensitive period of the infants’ engagement, we had to increase the research midwife’s attendance and involvement in the study as compared to what was initially expected. As we mentioned above (see Objective 1 for more details) the research midwife gave private sessions to the parents, i.e., helping mothers with feeding and soothing practices before, during and after the ABR recording process. Additionally, the CRS fully complied with the approved protocol and the project was officially arranged to be a part of the initial screening process the infants followed in the hospital. Therefore, all physicians attending the specific sample were informed about the study and integrated it in their necessary or suggested newborn protocols. This integration provided more safety feelings to the mothers, knowing that all responsible, caring persons knew about the study, while it also helped the research team to exclude possible confounding variables that could rise in due to the intervention.

Concerning the sufficiency of technology and equipment used for the collection, management, and analysis of data, they were available when needed. Equipment was stored and locked in the premises of the hospital, having a dedicated room for all consumables as well as the main ABR unit. Using a dedicated trolley, we set up all the equipment on it without moving it from that trolley. This way, we ensured a similar technical setup for all participants, while most importantly we avoided possible electricity interference coming from surrounding materials and other fitted to the room installations during the ABR data collection process as we could easily identify their electrical effect (see for example the breast pumps installation mentioned above). We also used a set of dedicated rooms to conduct the measurements. These places were very close to the dedicated (breast) feeding rooms in the neonatal ward. As an alternative, we were taking measures in specific low-electricity labor, delivery, and recovery rooms (LDR), using the practice of rooming-in. According to the World Health Organization and United Nations Children’s Fund, rooming-in is a “hospital practice where postnatal mothers and normal infants stay together in the same room for 24 h a day from the time they arrive in their room after delivery” [40]. This practice, which is greatly used in the UAE settings, helped us a little bit more to carry out our measurements, as mothers quickly learned their babies’ needs, how to care for, soothe, and comfort their newborns—i.e., rooming-in practice seems to promote early breastfeeding and encourage the maternal-infant bonding [41]. Also, in terms of the house visits mentioned before, we were ready to comply with the participants’ wishes, although we didn’t need to as no one asked for it.

Despite the deep clinical specialization and extended experience, the CRS entailed and clearly showcased throughout this stage of our study, it was decided that some extra research training was needed for them to be able to fully grasp the protocol concept and use the available equipment as expected. For this reason, the PI scheduled 4 + 4 h of training before and during the implementation of the study, offering training (a) to the collaborating audiologists on how to use, read and apply the specific ABR recording software in a research graded context (i.e., with the help of a volunteering infant that was later excluded from the study) and (b) on how the collaborating midwife, nurses and physicians should communicate the protocol design, administrative needs and the study’s background knowledge so as to maximize recruitment and retention. More specifically, the PI explained the test settings for ABR acquisition (i.e., how to set the stimulus type, duration, frequency, rate, polarity, intensity, and the number of sweeps) according to the study’s aims and goals, while he also gave clarifications regarding the threshold measurements for Air Conduction ABR, the interpretation of the ABR waveforms, the determination of thresholds, and how to deal with unusual or unexpected waveforms or results when experiencing them in the particular clinical setting. In terms of the consequent ABR signal analysis, although the initial plans were for the audiologists to perform this task, the PI decided to specifically train a research assistant (RA) for it. This way, more time could be dedicated from the audiologists on recording clearer data, while the RA could specifically work the analysis in parallel to the acquisition process. The PI trained the RA on the ABR data tabulation, pre-processing, processing, and signal analysis protocols, while conducting face-to-face and online sessions, demonstrating how to use the software with real data.

### 3.5. Preliminary Evaluation of Participant Responses to Intervention (Objective 5)

**Figure 1:**
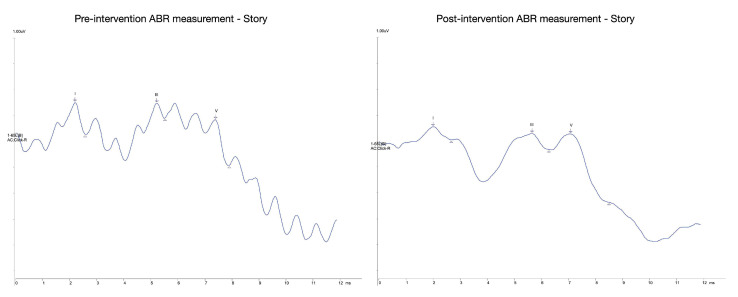
Averaged waveforms for the SEG (pre-post intervention).

**Figure 2:**
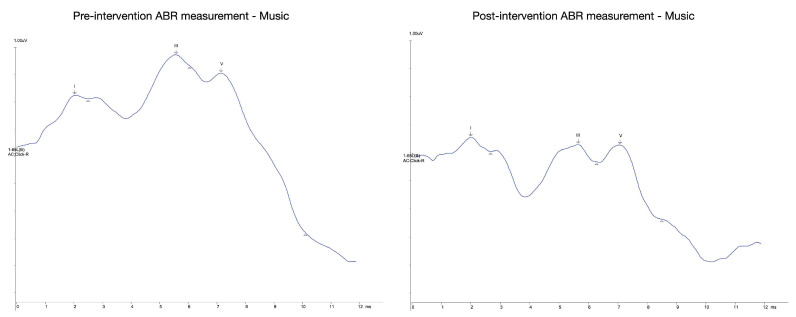
Averaged waveforms for the MEG (pre-post intervention).

The examination of the ABR data collected suggests that there are progressive effects brought about by both conditions of the interventions. However, the low number of sample prevents any preliminary comparative discussion. The figures and table below (Figure 1 and Figure 2 and Table 2) summarize the averaged ABR recordings of wave latencies for the music (MEG) and story (SEG) cohorts for the left ear. The outcomes evidently showed that there may be improvements in the latency and interpeak latency differences among the pre- and post-measurements for both cohorts. We also observed that the majority of the wave peaks (I, III, V) for both cohorts showed earlier latencies in their post-intervention measurement than their pre-intervention measurement. For example, our results showed that the latency range for wave V was obtained between 7.08 and 7.42 ms for both experimental groups. According to the literature, wave V is typically obtained between 6.0 and 9.0 ms at moderate stimulus levels (e.g., 65–75 dB nHL) [42]. Consequently, the V–I latency interval was recorded between 5.03 and 5.2 ms after the onset of the stimulus. Usually, a mean value of the V–I latency interval in full-term neonates is recorded at 5 ms (SD ± 0.3) [43]. Additionally, latencies for wave I were mainly obtained at 2.02 ms and for wave III at 5.2 ms for both averaged measurements. Our results rely upon the anticipated latencies of neonate ABR waveforms, as waves I and III are usually elicited at 2.0 and 4.8 ms (SD ± 0.3) respectively, by 70 dB nHL at a rate of 11/s [43].

In summary, the calculated averages demonstrated that the recordings gathered are significant and within the expected baseline of neonate ABR recordings. As already mentioned, the I–III–V peaks and interpeak intervals are potential points of observation for the study, as literature implies that developmental changes in latencies are mostly observable only after three months of age [22,44]. Most importantly, the outcomes evinced that the observed changes meet the expected direction of the study, potentially showcasing impacts that are beyond unprovoked normal maturation. We propose that the consistency of the recordings is enough to warrant that further development on the study protocols is not pertinent and therefore appropriately reliable.

## 4. Conclusions

For the purpose of the current study, we conducted a feasibility analysis on a ‘music versus language’ intervention design and its subsequent ABR measurements. This endeavor took place in an attempt to enhance our future steps, while fine-tuning our research project so as to provide a more detailed and reliable assessment of the participating newborns. At this stage, we focused mainly on our methodology, and assessed the fitness of our protocol through five specific objectives. The idea of assessing our study through these objectives is characterized by the fact that we wanted to avoid overlooking some risks concerning the protocol design, the data collection process and eligibility criteria for our participants. Completing the feasibility assessment process, it is essential to mention that the implementation of this specific analysis revealed some significant factors that we must manipulate before starting the main experimental stage.

Most importantly, our feasibility analysis revealed possible improvements in the application of our study communications as well as on the application of the ABR protocol. Additionally, our study helped us to understand and fully comprehend the importance of extra training of the mothers and collaborating physicians in an attempt to more precisely follow our protocol concept and the use of our available equipment. Furthermore, the change of the ABR protocol from multiple to just two-session appointments of recordings per day solidified our measurements process and gave stability to the sensitive recruitment-measurement balance we are called to follow in our future investigation steps. The specific feasibility analysis helped us also to re-examine our experimental ideas and avoid risk factors in our experimental design coming mainly from the multilingual framework the project encompasses and uses. It is proven now to us that extra care should be taken for projects running in multilingual environments to explain the recruitment process, participation, and follow-up steps. Specific participation requirements that focus on clinical interventions or interventions taking place in a clinical environment should be always explained at multiple levels and with multiple repetitions, even if participants seem to be comfortable and positive to their participation and processes asked to follow. Finally, the feasibility analysis confirmed our fitness to collect and analyze ABR data, supporting furthermore our initial hypothesis concerning the expected baseline of neonate ABR recordings. Hopefully our findings bring new insights for supporting music intervention as a fundamental factor for neurodevelopmental connectivity in full-time newborns and provide an informative design and assessment baseline for other researchers to rely on for their future study endeavors.

## Figures and Tables

**Table 1:** Number of delivered pregnancies in the selected study setting.

Total Amount of Pregnancies per Year	4000
Per month	300–350
Per week	70–80
Per day	10–11

**Table 2:** Summative averaged values of wave peaks I, III, V, and interpeak differences for the music and story cohort (AR = amplitude ratio).

	Left Ear						
	Peaks	I	III	V	III–I	V–III	V–I
Music Pre	Latency (ms)	2.05	5.6	7.17	3.55	1.58	5.13
	Amplitude (uV)	0.01	0.04	0.58	3.46 AR	13.73 AR	47.51 AR
Music Post	Latency (ms)	2.05	5.2	7.08	9.58	−4.83	5.03
	Amplitude (uV)	0.1	0.6	0.18			1.88 AR
Story Pre	Latency (ms)	2.23	5.25	7.42	3.02	2.17	5.2
	Amplitude (uV)	0.12	0.06	0.18	0.47 AR	3.05 AR	1.44 AR
Story Post	Latency (ms)	2.02	5.67	7.1	3.65	1.42	5.07
	Amplitude (uV)	0.05	0.06	0.26	1.24 AR	4.29 AR	5.33 AR

## Data Availability

You may find the collected datasets at the OsloMet web-cloud repository. Due to sensitive data, permission to access may be granted upon request. Please contact the corresponding author for access.

## Appendix A. Objectives and Guiding Questions for a Feasibility Study

Objective 1: Evaluation of Recruitment Capability and Resulting Sample Characteristics.

Main Question: Can we recruit appropriate participants?

1. How many potential eligible members of the targeted population are accessible in the local community?
2. What are the recruitment rates?
  - How many participants enter the study at a time?
  - How long does it take to recruit enough participants into the study?
  - What are the refusal rates for participation?
3. How feasible and suitable are eligibility criteria?
  - Are criteria clear and sufficient or too inclusive or restrictive?
4. What are the obstacles to recruitment?
  - Are colleagues and local organizations willing to assist with recruitment?
  - What are the reasons for refusal or ineligibility?
5. How relevant is the intervention to the intended population?
  - Do study participants show evidence of need for the intervention?
  - Are the characteristics of the study participants consistent with the range of expected characteristics as informed by the research literature?

Objective 2: Evaluation and Refinement of Data Collection Procedures and Outcome Measures.

Main Question: How appropriate are the data collection procedures and outcome measures for the intended population and purpose of the study?

1. How feasible and suitable are the data collection procedures?
  - Do participants understand the questions and other data collection procedures?
  - Do they respond with missing or unusable data?
2. How feasible and suitable is the amount of data collection?
  - Do the participants have the capacity to complete the data collection procedures?
  - Does the overall data collection plan involve a reasonable amount of time or does it create a burden for the participants?
3. Do the measures appear to be performing in a consistent way with the intended population as compared to measurement information available in the research literature?
  - Are internal consistency indicators of measures with the recruited sample congruent with expectations based on prior studies reported in the research literature?
  - Do planned outcome measures appear to be sensitive to the effects of the intervention?
  - Does a suitable outcome measure need to be developed?

Objective 3: Evaluation of Acceptability and Suitability of Intervention and Study Procedures.

Main Question: Are study procedures and intervention suitable for and acceptable to participants?

1. What are the retention and follow-up rates as the participants move through the study and intervention?
2. What are the adherence rates to study procedures, intervention attendance, and engagement?
  - Does the intervention fit with the daily life activities of study participants?
  - Do the participants have enough time and capacity to complete the intervention?
  - Does the intervention involve a reasonable amount of time, or does it create a burden for the participants?
  - To what extent is the intervention acceptable and appealing to participants?
  - If appropriate, how many participants agree to be randomized to group?
3. What is the level of safety of the procedures in the intervention?
  - Are there any unexpected adverse events?

Objective 4: Evaluation of Resources and Ability to Manage and Implement the Study and Intervention.

Main Question: Does the research team have the resources and ability to manage the study and intervention?

1. Does the research team have the administrative capacity, expertise, skills, space and time to conduct the study and intervention?
2. Can we conduct the study procedures and intervention in an ethical manner?
  - To what extent does staff comply with the approved human participants’ protocol?
  - How effectively are adverse events during implementation identified, documented, and reported?
3. Can the study and intervention be conducted within the designated budget?
4. Is the technology and equipment sufficient to conduct the study and intervention, including collection, management, and analysis of data?
  - Is equipment available when needed?
  - What is involved in training personal and/or participants to use the equipment?
5. Are we able to manage data entry and analysis efficiently and effectively?

Objective 5: Preliminary Evaluation of Participant Responses to Intervention.

Main Question: Does the intervention show promise of being successful with the intended population?

1. Does examination of quantitative data suggest that the intervention is likely to be successful?
  - Does examination of the data at the participant level suggest that changes in key outcome variables occurred?
  - Are the changes of the outcome variable(s) in the expected direction?
  - Do the estimates of effects suggest that the intervention has promise?
2. Do participants or relevant others provide qualitative feedback that may be indicative of the likelihood that the intervention will be successful?
3. If the quantitative and/or qualitative data suggest that the intervention is not promising:
  - Are the data collection procedures and outcome measures appropriate for the population and study?
  - Are the outcome measures and intervention theoretically aligned?
  - Is there evidence that the intervention does not produce change in the desired outcomes?
  - Is there evidence that the intervention was not implemented in the intended manner?
  - Have too many adaptations been made in the intervention process to adequately assess the participants’ responses to the intervention?
  - Are the findings congruent with the proposed theoretical model for the intervention?
