# Correction: Papatzikis et al. Key Challenges and Future Directions When Running Auditory Brainstem Response (ABR) Research Protocols with Newborns: A Music and Language EEG Feasibility Study. *Brain Sci*. 2021, *11*, 1562

**DOI:** 10.3390/brainsci15060606

**Published:** 2025-06-04

**Authors:** Efthymios Papatzikis, Mahmoud Elhalik, Shannaiah Aubrey Mae Inocencio, Maria Agapaki, Rosari Naveena Selvan, Faseela Shejeed Muhammed, Nazreen Abdulla Haroon, Swarup Kumar Dash, Maria Sofologi, Antonia Bezoni

**Affiliations:** 1 Department of Early Childhood Education and Care, Oslo Metropolitan University, 0167 Oslo, Norway; 2 Neonatal Intensive Care Unit, Latifa Women and Children’s Hospital, Dubai 9115, United Arab Emirates; mselhalik@dha.gov.ae (M.E.); fhvmuhammed@dha.gov.ae (F.S.M.); nazreenpharoon@gmail.com (N.A.H.); sdash@dha.gov.ae (S.K.D.); 3 Department of Psychology, Canadian University Dubai, Dubai 415053, United Arab Emirates; ishannaiah@gmail.com; 4 Independent Researcher, 71305 Iraklio, Greece; maria.agapaki94@gmail.com; 5 Institute for Physics 3—Biophysics and Bernstein Center for Computational Neuroscience, University of Göttingen, 37073 Göttingen, Germany; rselvan@uni-muenster.de; 6 Department of Psychology, University of Münster, 48149 Münster, Germany; 7 Psychology Laboratory, Department of Early Childhood Education, School of Education, University of Ioannina, 45110 Ioannina, Greece; m.sofologi@uoi.gr; 8 Institute of Humanities and Social Sciences, University Research Centre of Ioannina, 45110 Ioannina, Greece; 9 Department of Midwifery, Røyken Health Station, 3440 Røyken, Norway; toniampez@gmail.com

In the original publication [1], there was an inaccuracy in the description of the musical pieces used during the intervention. Specifically, we referred to the “baroque era” and “baroque music” rather than to the “classical era” and “classical music”. According to the article’s core methods, the newborns were exposed to two instrumental pieces by Mozart and Haydn, both of whom are central figures of the classical era. This incorrect labeling may lead to confusion about the musical repertoire employed. The corrected description now accurately states that the intervention involved classical-era compositions performed and recorded by professional musicians. This change does not affect the study’s overall findings or conclusions but ensures precision in classifying the music used in the intervention and aligns the written text with the original intent and actual musical content. The corrected text appears below in bold font.

The newborns were randomly divided into two experimental groups, following a music and a storytelling intervention. No control group could be recruited at this stage as originally designed, due to the COVID-19 outbreak as mentioned above. Both experimental groups were ABR measured pre and post the intervention. Starting right after the first 24 h after delivery, the intervention was taking place for seven consecutive days, twice per day during any two of the daily breastfeeding/feeding episodes. For the music experiment group (MEG), we used two purely instrumental musical pieces coming from the **classical** era by Mozart and Haydn. The use of **classical** music was based on the assumption that this type of music was not typically accessible to newborns in the region to be exposed to, hence reducing the degree of exogenous to the protocol exposure as a confounding variable. The two music pieces were professionally recorded and performed by musicians. Their overall duration was 20 min. The pieces were compressed to an mp3 high bit-rate format for easier distribution through a mobile phone application. They were pre-processed and re-mastered to normalize and equalize their sound output and their frequency distribution below and up a certain frequency threshold (High Pass: 15 Hz—Low Pass: 8 kHz) to achieve sound consistency for all mobile phone speakers. They were also balanced at a 120-bpm rate.

The authors state that the scientific conclusions are unaffected. This correction was approved by the Academic Editor. The original publication has also been updated.

## References

[1] Papatzikis E., Elhalik M., Inocencio S.A.M., Agapaki M., Selvan R.N., Muhammed F.S., Haroon N.A., Dash S.K., Sofologi M., Bezoni A. (2021). Key challenges and future directions when running auditory brainstem response (ABR) research protocols with newborns: A music and language EEG feasibility study. Brain Sci..

